# Interferon-Gamma Modulation of the Local T Cell Response to Alphavirus Encephalomyelitis

**DOI:** 10.3390/v12010113

**Published:** 2020-01-16

**Authors:** Victoria K. Baxter, Diane E. Griffin

**Affiliations:** 1 W. Harry Feinstone Department of Molecular Microbiology and Immunology, Johns Hopkins Bloomberg School of Public Health, Baltimore, MD 21205, USA; vkbaxter@email.unc.edu; 2 Department of Molecular and Comparative Pathobiology, Johns Hopkins University School of Medicine, Baltimore, MD 21205, USA

**Keywords:** Sindbis virus, viral encephalomyelitis, viral RNA clearance, interferon-gamma, T cells, NK cells, granzyme B

## Abstract

Infection of mice with Sindbis virus (SINV) provides a model for examining the role of the immune response to alphavirus infection of the central nervous system (CNS). Interferon-gamma (IFN-γ) is an important component of this response, and we show that SINV-infected differentiated neurons respond to IFN-γ in vitro by induction of antiviral genes and suppression of virus replication. To determine the in vivo effects of IFN-γ on SINV clearance and T cell responses, C57BL/6 mice lacking IFN-γ or IFN-γ receptor-1 were compared to wild-type (WT) mice after intracranial SINV infection. In WT mice, IFN-γ was first produced in the CNS by natural killer cells and then by CD4^+^ and CD8^+^ T cells. Mice with impaired IFN-γ signaling initiated clearance of viral RNA earlier than WT mice associated with CNS entry of more granzyme B-producing CD8^+^ T cells. However, these mice established fewer CD8^+^ tissue-resident memory T (T_RM_) cells and were more likely to experience reactivation of viral RNA synthesis late after infection. Therefore, IFN-γ suppresses the local development of granzyme B-expressing CD8^+^ T cells and slows viral RNA clearance but promotes CD8^+^ T_RM_ cell establishment.

## 1. Introduction

Response to viral infections of the central nervous system (CNS) poses a unique problem for the immune system. The restrictive nature of the blood–brain barrier limits the ability of proteins and immune cells to enter into the brain and spinal cord in response to a virus infection [1]. Resident cells of the CNS, particularly neurons, have a limited capacity to express major histocompatibility complex (MHC) molecules [2,3]. Because neurons are a valuable but finite and minimally renewable cell population, preservation of neuronal function requires that infected neurons be allowed to survive, necessitating noncytolytic immune mechanisms to control virus infection.

Sindbis virus (SINV) is the prototypic member of the alphaviruses, a genus of enveloped, positive-sense, single-stranded RNA viruses belonging to the *Togaviridae* family [4]. SINV is neurotropic in mice, and when mice are infected with a strain of SINV that does not cause fatal encephalomyelitis (e.g*.,* TE), clearance of infection from the CNS occurs in three phases [5]. In Phase 1, during the first 7 to 8 days post infection (DPI), both infectious virus and viral RNA increase rapidly, followed by clearance of infectious virus that occurs primarily through cooperative effects of anti-SINV antibody and the cytokine interferon-gamma (IFN-γ) [6,7,8,9]. In Phase 2, from approximately 10 to 60 days, infectious virus is no longer recoverable, but declining levels of viral RNA are still readily detectable. Finally, in Phase 3, from 60 days through at least a year and presumably for the life of the animal, viral RNA persists at a low level [10,11].

The immune response to alphavirus infection in the CNS presents a double-edged sword: while local production of antibody and IFN-γ clears infectious virus [6,7,8,9], T cell-mediated inflammation is responsible for many of the pathological changes and much of the neurological damage produced [12,13,14]. CD8^+^ T cells participate in clearance of viral RNA because CD8 and B2m knockout mice clear viral RNA more slowly from the brains and spinal cords than wild-type (WT) mice [3]. 

IFN-γ, an important product of natural killer (NK) cells and CD4^+^ and CD8^+^ T cells, exerts its antiviral effects by inducing IFN-stimulated genes (ISGs), but also by modulating the immune response to infection. The IFN-γ receptor is a heterotetramer of ligand-binding IFN-γR1 and signaling IFN-γR2 expressed on many cell types, including neurons [15]. IFN-γ binding to its receptor triggers a Jak/Stat signaling pathway that can induce expression of over 200 ISGs, some of which have direct antiviral activity, while others modulate the immune response [15,16,17]. IFN-γ is particularly important for clearance of infectious virus from spinal cord neurons [7]. IFN-γ-induced immunomodulatory effects include immune cell activation, trafficking, and differentiation, as well as more direct intracellular antiviral activities [18]. 

IFN-γ is detectable in the CNS within 3 days after infection, peaks at 7 days and becomes undetectable by 10–14 days, although IFN-γ mRNA remains elevated for months [9]. Previous studies have shown that mice with impaired IFN-γ signaling produce lower levels of inflammatory cytokines and chemokines in the CNS, resulting in better food intake and less weight loss than their WT counterparts, but entry of fewer antibody-secreting cells, slower clearance of infectious virus and more reactivation of infectious virus 18 to 21 days after infection [6,7,8,9]. In the current study, we have further defined the role of IFN-γ by analyzing the in vitro role of IFN-γ in regulating SINV replication and clearance in differentiated neurons, identifying the sources of IFN-γ in the CNS of SINV-infected mice, and determining its role in clearance of SINV RNA and modulation of the T cell response. We show that IFN-γ induced an antiviral response in neurons in vitro with enhanced viral RNA clearance. In SINV-infected WT mice, IFN-γ was produced in the CNS by NK cells and T cells and inhibited development of the local CD8^+^ T cell response, resulting in slower clearance of viral RNA than in *Ifng*^−/−^ and *Ifngr1*^−/−^ mice. Understanding how the immune response is able to clear both infectious virus and viral RNA while simultaneously controlling reactivation may inform development of interventions.

## 2. Materials and Methods

### 2.1. Cell Cultures

AP-7 cells, a gift from Dale Hunter (Tufts University), were derived from rat olfactory neurons and immortalized with a temperature-sensitive simian virus-40 T antigen [19]. Cycling AP-7 (cAP-7) cells were grown at 33 °C with 7% CO_2_ in DMEM + 10% FBS + 2 mM l-glutamine + 100 U/mL penicillin + 100 μg/mL streptomycin. For differentiation, cAP-7 cells were transferred to 39 °C with 5% CO_2_ in DMEM + 10% FBS + 2 mM l-glutamine + 100 U/mL penicillin + 100 μg/mL streptomycin + 1 μg/mL insulin + 20 μM dopamine + 100 μM ascorbic acid for 5 to 7 days (dAP-7 cells).

### 2.2. Virus and Infection of Cells

The TE strain of SINV [20] was grown and assayed by plaque formation in BHK cells. cAP-7 or dAP-7 cells were infected with SINV TE for 1 h in DMEM + 1% FBS at a multiplicity of infection (MOI) of 1 or 5. dAP-7 cells were treated for 1 h with 500 U/mL recombinant rat IFN-γ (PBL Interferon Source) in DMEM + 1% FBS at 2 h before infection (IFN-γ Pre-Txt), 2 hours post infection (HPI, IFN-γ Txt 2 HPI), or 24 HPI (IFN-γ Txt 24 HPI). Supernatant fluids for plaque assays and cell pellets for protein and RNA analysis were collected in triplicate at indicated time points following infection and stored at −80 °C. Infectious virus was quantified by plaque assay on BHK cells at 37 °C, 5% CO_2_ for 48 h. Plaques were counted using 10% neutral red in PBS for visualization.

### 2.3. Virus Infection of Mice

Four to six week-old male and female wild-type C57BL/6 (WT) mice, mice deficient in IFN-γ receptor 1 (*Ifngr1*^−/−^*,* strain B6.129S7-*Ifngr1^tm1Agt^*/J, Jackson Labs), and mice deficient in IFN-γ (*Ifng*^−/−^*,* strain B6.129S7-*Ifng^tm1Ts^*/J, Jackson Labs) were intracranially (IC) inoculated with 10^3^ plaque forming units (pfu) of the TE strain of SINV diluted in 20 μL PBS or 20 μL PBS vehicle while under light isoflurane anesthesia. For fresh tissue collection, mice were euthanized by an overdose of isoflurane anesthesia, perfused with ice-cold PBS, and cervical lymph nodes (CLNs), brains, and/or spinal cords were collected. The Johns Hopkins University Institutional Animal Care and Use Committee approved protocols for all studies performed (MO15H160 approved 06/14/2015; MO18H178 approved 06/13/2018).

### 2.4. Immunoblot Analysis

For preparation of lysates, dAP-7 cells pooled from three wells were incubated on ice for 30 min in RIPA buffer (50 mM Tris, 150 mM NaCl, 1% SDS, 1% NP-40, 0.5% Na-deoxycholate, 1 mM EDTA) and centrifuged at 12,000 rpm for 15 min. Total protein was quantified by DC Protein assay (Bio-Rad, Hercules, CA, USA) using a BSA standard curve, and 10 μg was boiled in 6X SDS loading buffer (0.5 M Tris (pH 6.8), 30% glycerol, 10% SDS, 0.12% bromophenol blue, 6% β-mercaptoethanol) for 5 min. Samples were run on a 10% SDS-polyacrylamide gel electrophoresis (PAGE) gel and transferred to a nitrocellulose membrane (Bio-Rad). Membranes were blocked in TBS-0.1% Tween-20 (TBST) + 5% milk for 1 h at room temperature on a rocker and incubated overnight at 4 °C on a rocker with primary antibody diluted in TBST + 5% BSA (1:2000 rabbit polyclonal anti-nsp3; 1:10,000 rabbit polyclonal NSV anti-sera; 1:10,000 mouse monoclonal anti-β-actin, (Millipore, Burlington, MA, USA) [21,22]. Membranes were incubated with secondary antibody diluted in TBST + 5% nonfat milk (1:5000 horseradish peroxidase (HRP)-conjugated donkey anti-rabbit IgG for nsp3 and poly-NSV; 1:5000 HRP-conjugated sheep anti-mouse IgG for β-actin, GE Healthcare) for 1 h on a rocker and developed using Amersham ECL Western Blotting Detection Reagent (GE Healthcare) according to manufacturer’s instructions.

### 2.5. Gene Expression Measurement by Real-Time PCR

RNA was isolated from dAP-7 cells using the Qiagen RNeasy (Germantown, MD, USA) or RNeasy Plus Mini kit following the manufacturer’s directions. For mouse CNS tissue, right brain halves or whole spinal cords were homogenized in one mL QIAzol in Lysing Matrix D tubes (MP Biomedicals, Irvine, CA, USA) at 6.0 M/s for 40 s using a FastPrep-24 homogenizer (MP Biomedicals). The Qiagen RNeasy Lipid Tissue Mini kit was used to isolate RNA, and cDNA was synthesized using a High Capacity cDNA Reverse Transcription Kit with random primers (Life Technologies, Carlsbad, CA, USA), and quantitative real-time PCR (qRT-PCR) was performed using TaqMan Universal PCR Master Mix (Roche, Indianapolis, IN, USA) on a 7500 Fast Real-Time PCR System. SINV RNA copies were measured using TaqMan probe (5′-6-carboxyfluorescein (FAM)-CGCATACAGACTTCCGCCCAGT-6-carboxytetra-methylrhodamine (TAMRA)-3′, Applied Biosystems, Waltham, MA, USA) with primers to the SINV E2 gene (forward, 5′-TGGGACGAAGCGGACGATAA-3′; reverse, 5′-CTGCTCCGCTTTGGTCGTAT-3′). SINV E2 copies were quantified using a standard curve made of ten-fold dilutions of a plasmid containing the SINV subgenomic region genes and normalized to endogenous rodent *Gapdh*. mRNA was measured using commercially available TaqMan gene expression assays (Applied Biosystems or Integrated DNA Technologies, Coralville, IA, USA), and relative quantification was performed by the ΔΔCT method using endogenous rodent *Gapdh* mRNA for normalization.

### 2.6. Mononuclear Cell Isolation

Single cell suspensions were made from CLNs, brains, and spinal cords pooled from 3 to 9 mice per strain per time point as previously described [9]. Briefly, CLNs were dissociated using gentleMACS C tubes and Dissociator (Miltenyi Biotech, Auburn, CA, USA), and red blood cells were lysed with an ammonium chloride solution (Sigma-Aldrich, St. Louis, MO, USA or eBioscience, San Diego, CA, USA). Brains and spinal cords were dissociated in a solution containing collagenase D (Roche) or collagenase IV (Worthington Labs, Worthington, OH, USA) and DNase I (Roche), and mononuclear cells were separated on a supplemented Percoll gradient. Live mononuclear cells were quantified using trypan blue exclusion.

### 2.7. Flow Cytometry

10^6^ live cells were stained with violet LIVE/DEAD Fixable Dead Cell Stain (Life Technologies) for 30 min, followed by a 15 min incubation with anti-mouse CD16/CD32 (BD Pharmingen) to block Fc receptors. Cells were stained with monoclonal antibodies against CD45 (clone 30-F11), CD3 (clone 17A2), CD4 (clone RM4-5), CD8 (clone 53-6.7), CD11b (clone M1/70), Ly6G (clone 1A8), Ly6C (clone HK1.4), NK1.1 (clone PK136), CD25 (clone PC61.5), CD44 (clone IM7), CD62L (clone MEL-14), and CD103 (clone 2E7), from eBioscience or BD Pharmingen diluted in PBS + 2 mM EDTA + 0.5% BSA (FACS Buffer) for 30 min on ice. For intracellular staining of Foxp3, cells were fixed for 20 min using Fixation/Permeabilization solution from the eBioscience Foxp3/Transcription Factor Staining Buffer kit. Cells were stained for 30 min on ice with a monoclonal antibody against Foxp3 (clone FKJ-16S, BD Pharmingen) diluted in Permeabilization Buffer.

For degranulation assessment, 2–3 × 10^6^ cells were stimulated for 4 h at 37 °C with 50 ng/mL of phorbol-12-myristate 13-acetate (PMA, Sigma), 1 μg/mL ionomycin (Sigma), and antibody against CD107a (clone eBio1D4B, eBioscience) in RPMI + 10% FBS. After 1 h, monensin (GolgiStop, 1:2000, BD Pharmingen, Franklin Lakes, NJ, USA) was added to block cellular protein transport. For intracytoplasmic cytokine staining (ICS), 2–3 × 10^6^ cells were stimulated for 4 h at 37 °C with 50 ng/mL PMA and 1 μg/mL ionomycin in the presence of brefeldin A (GolgiPlug, BD Pharmingen) in RPMI + 1% FBS. Following LIVE/DEAD and surface antibody staining (see above), cells were fixed for 20 min using Fixation/Permeabilization solution from the BD Cytofix/Cytoperm kit. Cells were stained for 30 min on ice with monoclonal antibodies against IFN-γ (clone XMG1.2), IL-4 (clone 11B11), IL-17a (clone eBio17B7), granzyme B (clone NGZB), granzyme A (clone GzA-3G8.5), GM-CSF (clone MPI-22E9), and TNF-α (clone MP6-XT22) from Ebioscience or BD Pharmingen diluted in BD Perm/Wash buffer.

Cells were resuspended in 200 μL FACS Buffer and run on a BD FACSCanto II cytometer using BD FACSDiva software, version 8, and analyses were carried out using FlowJo software, version 8. Cells were characterized as follows: CD4 T cells (CD45^hi^CD3^+^CD4^+^), CD8 T cells (CD45^hi^CD3^+^CD8^+^), NK cells (CD45^+^CD3^−^NK1.1^+^), macrophages (CD45^hi^CD11b^+^Ly6G^−^Ly6C^+^), neutrophils (CD45^+^CD11b^+^Ly6G^+^Ly6C^int^), microglia (CD45^lo^CD11b^+^Ly6G^−^Ly6C^−^), Th1 cells (CD3^+^CD4^+^IFN-γ^+^), Th2 cells (CD3^+^CD4^+^IL-4^+^), Th17 cells (CD3^+^CD4^+^IL-17a^+^), Tregs (CD3^+^CD4^+^CD25^+^Foxp3^+^), and tissue resident memory T (T_RM_) cells (CD44^hi^CD62L^−^CD103^+^). All flow cytometry data are presented as averages of 3 to 5 independent experiments.

### 2.8. Brain and Spinal Cord Histology

Following euthanasia, mice were perfused with ice-cold 4% paraformaldehyde (PFA), and brains and spinal columns were collected. Brains were cut into three coronal sections using an Adult Mouse Brain Slicer (Zivic Instruments, Pittsburgh, PA, USA), fixed overnight in 4% PFA, and embedded in paraffin. Spinal columns were fixed overnight in 4% PFA, decalcified for 36 h in a 10% sodium citrate/22% formic acid solution, cut to isolate the L4–L6 spinal cord regions, and embedded in paraffin.

10 μm tissue sections from 3 to 4 mice per group were stained with hematoxylin and eosin (H&E). Brain slides were coded and sections scored as previously described [23] using a 0–3(4) scale: 0, no detectable inflammation; 1, one or two small inflammatory foci; 2, moderate inflammatory foci in up to 50% of 10× magnification fields per section; 3, moderate to large inflammatory foci in greater than 50% of 10× magnification fields. An additional point was given for excessive parenchymal cellularity, allowing for a maximum score of 4. Spinal cord slides were coded and sections scored using a modified 0–2(3) scale as previously described [24]: 0, no detectable inflammation; 1, one to two small inflammatory foci; 2, greater than two inflammatory foci per spinal cord or moderate to marked inflammatory foci. An additional point was given for excessive parenchymal cellularity, allowing for a maximum score of 3.

### 2.9. Statistics

Statistical analyses were performed using Graphpad Prism 6 software. Time-course studies were analyzed by two-way ANOVA with Bonferroni’s or Tukey’s multiple comparison post-test for two group and three group comparisons, respectively. Comparisons between three groups at a single time point were made using one-way ANOVA with Tukey’s multiple comparisons post-test. A *p* value of <0.05 was considered significant for all analyses.

## 3. Results

### 3.1. IFN-γ Facilitates Virus Clearance from Neurons In Vitro

**Figure 1:**
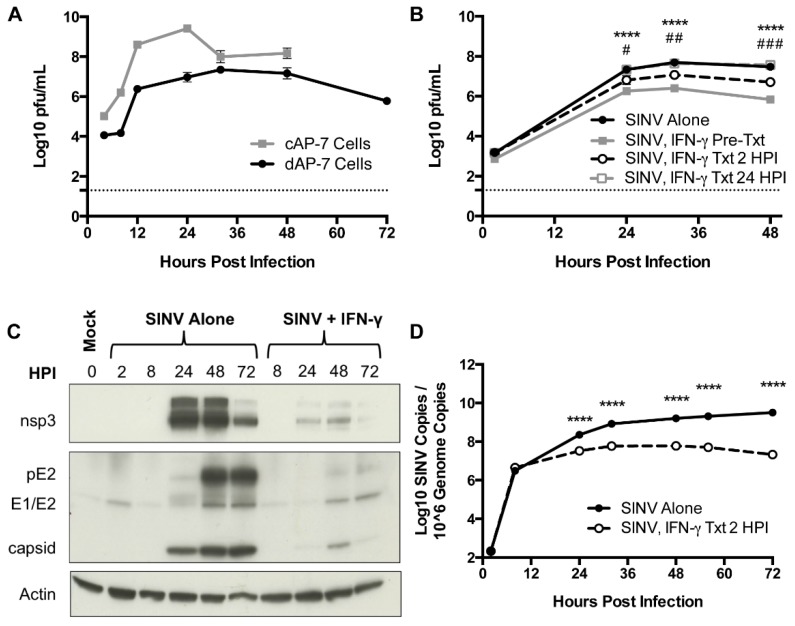
SINV replication in neurons in vitro and the effect of IFN-γ. (**A**) Infectious virus was measured by plaque assay after infection of cAP-7 (gray line) and dAP-7 (black line) cells infected with SINV TE at a MOI of 5. (**B**) dAP-7 cells were infected with SINV TE at a MOI of 1 and treated with 500 U/mL IFN-γ 2 h prior to infection (gray square and solid line; SINV, IFN-γ Pre-Txt), at 2 HPI (white circle and black dashed line; SINV, IFN-γ Txt 2 HPI), at 24 HPI (white square and gray dashed line; SINV, INF-γ Txt 24 HPI) or untreated (black circle and solid line; SINV Alone) (*n* = 3 replicates per cell type per treatment group; data are representative of two (**A**) or three (**B**) independent experiments and are presented as the mean ± SEM; dashed line indicates level of detection; **** *p <* 0.0001, SINV Alone vs. SINV, IFN-γ Pre-Txt; ^#^ *p <* 0.05, ^##^ *p <* 0.01, ^###^ *p <* 0.001, SINV Alone vs. SINV, IFN-γ Txt 2 HPI by Tukey’s multiple comparisons test). (**C**,**D**) dAP-7 cells were infected with SINV at a MOI of 1 and either left untreated or treated with 500 U/mL IFN-γ at 2 HPI. (**C**) Nonstructural (nsp3) and structural (pE2, E1/E2, capsid) SINV protein production was evaluated by western blot using β actin as control. (**D**) Viral RNA levels were evaluated by qPCR in untreated (black circle and solid line) and IFN-γ-treated (white circle and black dashed line) dAP-7 cells (*n* = 3 replicates per treatment group; data are representative of two independent experiments and are presented as the mean ± SEM; **** *p <* 0.0001, by Bonferroni’s multiple comparisons test).

To elucidate a potential role for IFN-γ in virus clearance from neurons, immature cycling cAP-7 olfactory neuronal cells and mature bipolar differentiated dAP-7 cells [19] were infected with SINV (MOI = 5). Both cAP-7 and dAP-7 cells supported virus replication, with cAP-7 cells producing higher peak titers than dAP-7 cells and dying by 48 HPI (Figure 1A). dAP-7 cells continued to produce virus through 72 HPI and were used for subsequent studies of the effect of IFN-γ on virus replication.

dAP-7 cells infected with SINV (MOI = 1) were treated with 500 U/mL rat recombinant IFN-γ 2 h before infection, 2 HPI, or at 24 HPI. Virus replicated in all treatment groups, with titers peaking at about 32 HPI (Figure 1B). dAP-7 cells treated with IFN-γ prior to infection and at 2 HPI had significantly decreased virus production compared to untreated cells at 24, 32, and 48 HPI, with the greatest effect seen in pretreated cells, demonstrating the ability of neurons to develop an IFN-γ-induced antiviral response. Treatment with IFN-γ at 24 HPI did not alter virus production.

To assess the effect of IFN-γ on virus replication after infection was established, production of SINV proteins was examined by immunoblot in dAP-7 cells infected with SINV alone or treated with IFN-γ at 2 HPI. In untreated SINV-infected cells, production of the nonstructural nsp3 protein and structural capsid protein reached high levels by 24 HPI (Figure 1C). Production of the E1 and E2 structural glycoproteins (along with precursor to E2, pE2) was evident by 48 HPI and diminished by treatment with IFN-γ. These results show that IFN-γ can decrease the production of SINV by neurons.

We next sought to determine the effects of IFN-γ on production of viral RNA. SINV-infected dAP-7 cells were treated with IFN-γ at 2 HPI, and cell pellets collected to quantify viral RNA by qRT-PCR. Viral RNA copies were comparable at 8 HPI. Copy number in untreated cells continued to rise, peaking at 72 HPI, while SINV RNA levels plateaued in treated cells and by 72 HPI had begun to decrease (Figure 1D). Overall, viral RNA synthesis was significantly inhibited by IFN-γ treatment compared to untreated dAP-7 cells from 24 to 72 HPI. These studies show that IFN-γ signaling affects production and clearance of both infectious virus and viral RNA from neurons in vitro.

### 3.2. IFN-γ Induces Neuronal Expression of Antiviral ISGs

**Figure 2:**
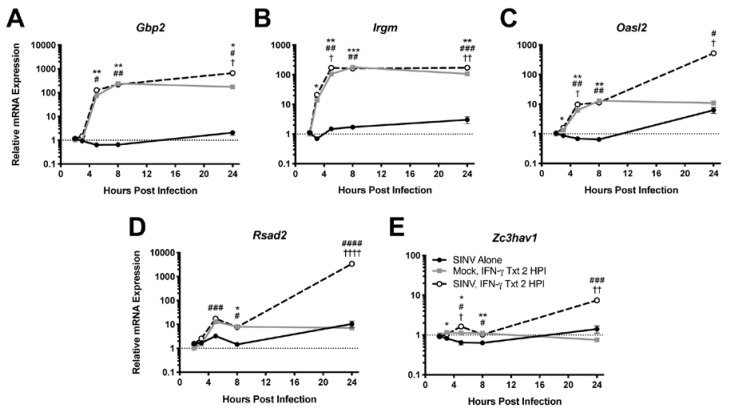
Effect of IFN-γ treatment on ISG expression during SINV infection in vitro. ISGs selected for examination by qRT-PCR in dAP-7 cells infected with SINV at a MOI of 1 and left untreated (black circle and solid line), mock-infected and treated with 500 U/mL IFN-γ at 2 HPI (gray square and solid line), or SINV-infected at a MOI of 1 and treated with 500 U/mL IFN-γ at 2 HPI (white circle and black dashed line) included *Gbp2* (**A**), *Irgm* (**B**), *Oasl2* (**C**), *Rsad2* (**D**), and *Zc3hav1* (**E**) (*n* = 3 replicates per group; data are representative of two independent experiments and are presented as the mean ± SEM; dashed line indicates gene expression of untreated, mock-infected cells to which other groups were normalized; * *p <* 0.05, ** *p <* 0.01, *** *p <* 0.001, SINV Alone vs. Mock, IFN-γ Txt 2 HPI; ^#^ *p <* 0.05, ^##^ *p <* 0.01, ^###^ *p <* 0.001, ^####^ *p <* 0.0001, SINV Alone vs. SINV, IFN-γ Txt 2 HPI; ^†^ *p <* 0.05, ^††^ *p <* 0.01, ^††††^ *p <* 0.0001, Mock, IFN-γ Txt 2 HPI vs. SINV, IFN-γ Txt 2 HPI, all by Tukey’s multiple comparisons test).

Because IFN-γ facilitates virus clearance from neurons, we examined induction of antiviral genes by IFN-γ. mRNA expression of representative antiviral ISGs was examined by qRT-PCR in dAP-7 cells infected with SINV (MOI = 1) and treated with 500 U/mL IFN-γ at 2 HPI. *Gbp2* (Figure 2A) and *Irgm* (Figure 2B), two genes associated with autophagy [25], were highly expressed by SINV-infected dAP-7 cells treated with IFN-γ, as were *Oasl2* (Figure 2C), a member of the 2’-5’oligoadenylate/RNaseL system [26], and *Rsad2* (Figure 2D), which encodes viperin, a protein that interferes with assembly and release of many viruses [27]. *Zc3hav1* (Figure 2E), which encodes ZAP/PARP13, a protein involved in viral RNA degradation and induction of the innate immune response that restricts alphavirus and flavivirus replication [28,29], was less highly upregulated. All of these genes required IFN-γ for induction and generally were not induced by SINV infection alone. 

### 3.3. Source of IFN-γ during In Vivo SINV CNS Infection

**Figure 3:**
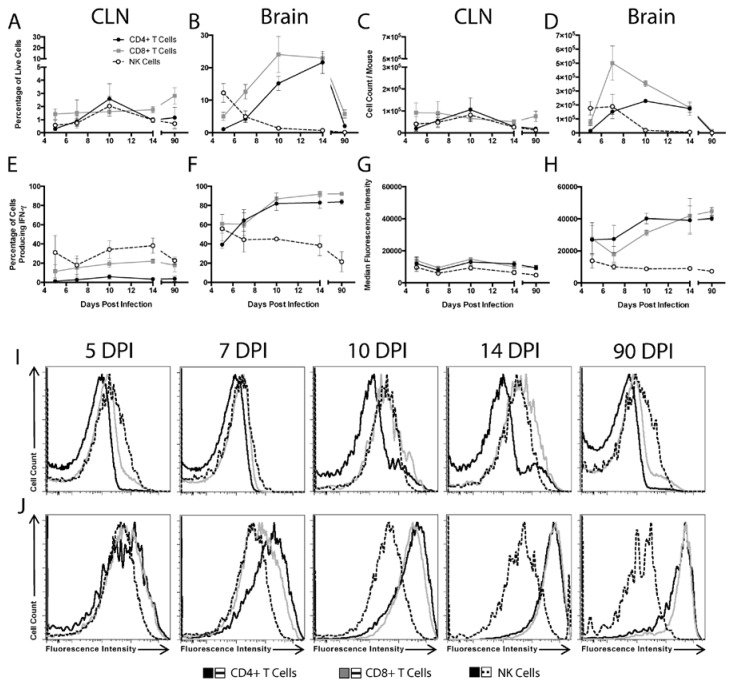
Source of IFN-γ during SINV infection. Flow cytometry was used to evaluate what cells were producing IFN-γ in the CLNs (**A**,**C**,**E**,**G**,**I**) and brains (**B**,**D**,**F**,**H**,**J**) of WT mice at 5, 7, 10, 14 and 90 DPI. CD4^+^ T cells (black circle and solid line), CD8^+^ T cells (gray square and solid line), and NK cells (white circle and black dashed line) producing IFN-γ were examined as both the percentage of live cells (**A**,**B**) and absolute cell counts (**C**,**D**). Also evaluated were the percentage of each cell type producing IFN-γ (E, F) and the MFI of IFN-γ for each cell type presented in graph form (**G**,**H**) and as histograms (**I**,**J**) (*n* = 3–7 pooled mice per time point from three independent experiments, except for data from 5 DPI CLNs, which were from two independent experiments; data are presented as the mean ± SEM).

To characterize the time course and source of IFN-γ throughout the course of SINV infection of the CNS, flow cytometry was used to characterize cells from the CLNs, the draining lymph nodes of the brain, and the brains of WT mice (Figure 3). CD4^+^ T cells, CD8^+^ T cells, and NK cells were examined for cytokine production during Phase 1 (5 and 7 DPI), Phase 2 (10 and 14 DPI), and Phase 3 (90 DPI) of infection. In the CLNs, few cells produced IFN-γ at any time (Figure 3A,C). In the brain, NK cells were the predominant source of IFN-γ at 5 DPI, both as percentage of live cells and absolute numbers (Figure 3B,D). Numbers of IFN-γ-producing cells in the brain peaked at 7 DPI and were predominantly CD8^+^ T cells. As CD8^+^ T cells decreased, CD4^+^ T cells became comparable contributors by 14 DPI. In Phase 3 of infection, fewer T cells were present in the brains and CD8^+^ and CD4^+^ T cells produced the majority of IFN-γ. Therefore, NK cells produce most of the local CNS IFN-γ early in the course of infection, but at later times, CD4^+^, and especially CD8^+^, T cells become the predominant source.

We next characterized the percentages of each cell population producing IFN-γ. Little change was seen in the CLNs, with less than 10% of CD4^+^ T cells, less than 20% of CD8^+^ T cells, and 20–40% of NK cells producing IFN-γ at any time after infection (Figure 3E). In the brain, the percentage of CD4^+^ and CD8^+^ T cells producing IFN-γ increased over the course of infection, going from approximately 40 to 60% at 5 and 7 DPI to over 80% at 10, 14, and 90 DPI (Figure 3F). In contrast, the percentage of brain NK cells producing IFN-γ remained between 20 to 50%, similar to that in the CLNs.

To compare the relative amounts of IFN-γ produced by each cell type, median fluorescence intensities (MFIs) were determined. In the CLNs, the MFI for IFN-γ remained low and did not change for any cell population (Figure 3G,I). However, in the brain, the IFN-γ MFIs for CD4^+^ and CD8^+^ T cells, but not NK cells, increased over time, with the greatest increase occurring between 7 and 10 DPI (Figure 3H,J).

### 3.4. Effect of IFN-γ Signaling on ISG Expression in the CNS of SINV-Infected Mice

**Figure 4:**
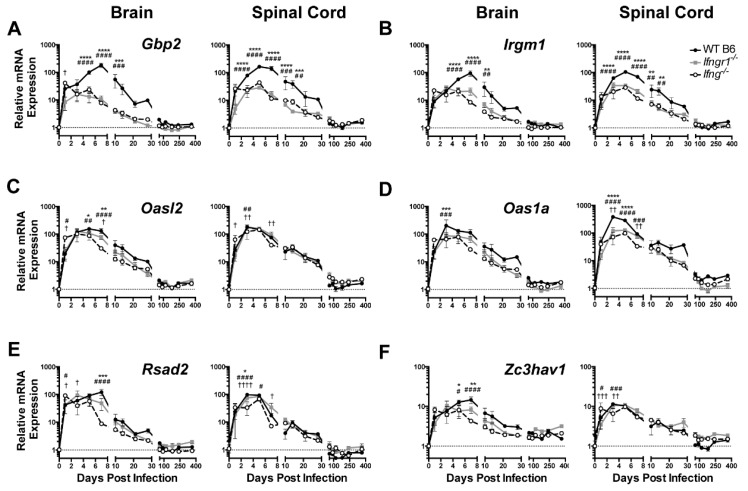
ISG expression in SINV-infected mice with impaired IFN-γ signaling. Expression of *Gbp2* (**A**), *Irgm1* (**B**), *Oasl2* (**C**), *Oas1a* (**D**), *Rsad2* (**E**), and *Zc3hav1* (**F**) were examined by qRT-PCR in the brains (**left panels**) and spinal cords (**right panels**) of WT (black circle and solid line), *Ifngr1^−/−^* (gray square and solid line), and *Ifng^−/−^* (white circle and black dashed line) mice (*n* = 3–6 mice per strain per time point; data are presented as the mean ± SEM; dashed line indicates gene expression of 0 DPI tissue for each strain to which other time points were normalized; * *p <* 0.05, ** *p <* 0.01, *** *p <* 0.001, **** *p <* 0.0001, WT vs. *Ifngr1^−/−^*; ^#^ *p <* 0.05, ^##^ *p <* 0.01, ^###^ *p <* 0.001, ^####^ *p <* 0.0001 WT vs. *Ifng^−/−^*; ^†^ *p <* 0.05, ^††^ *p <* 0.01, ^†††^ *p <* 0.001, ^††††^ *p <* 0.0001, and *Ifngr1^−/−^* vs. *Ifng^−/−^*, all by Tukey’s multiple comparisons test).

To determine the in vivo role of IFN-γ during SINV encephalomyelitis, the responses of mice deficient in IFN-γ (*Ifng^−/−^)* or in the α-chain of the IFN-γ receptor (*Ifngr1^−/−^)* were compared to those of WT mice. To assess expression of antiviral ISG mRNAs for the five antiviral ISGs previously selected for in vitro analysis of neuronal responses plus *Oas1a*, a protein associated with the 2’-5’oligoadenylate/RNase L system that is more active than *Oasl2* in mice [26] were examined by qRT-PCR (Figure 4). *Gbp2*, *Irgm1*, *Oasl2*, *Rsad2*, and *Zc3hav1* were up regulated in the brains and spinal cords of all mice during SINV infection, likely reflecting the overlap with ISGs induced by type I IFN. However, expression of *Gbp2* (Figure 4A) and *Irgm1* (Figure 4B) at 5-10 DPI in the brain and spinal cord were higher in WT mice than *Ifngr1^−/−^* and *Ifng^−/−^* mice. Smaller differences in expression levels in brain were seen at day 5 and 7 for *Oasl2* (Figure 4C) and at 3 DPI *Oas1a* (Figure 4D) and at 7 DPI for *Rsad2* (Figure 4E) and *Zc3hav1* (Figure 4F). These results show that while ISGs are induced during SINV infection in the CNS of mice with impaired IFN-γ signaling, expression is diminished compared to that of mice with intact IFN-γ signaling.

### 3.5. Effect of IFN-γ Signaling on Viral RNA Clearance from the CNS of SINV-Infected Mice

**Figure 5:**
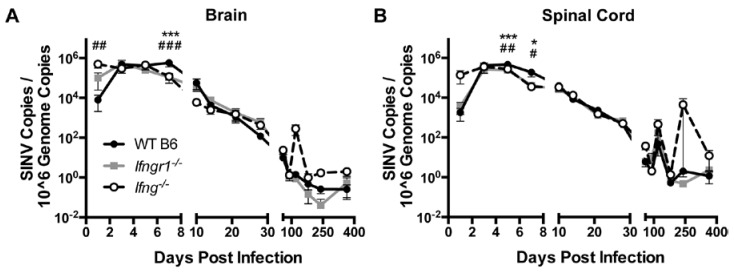
Effect of IFN-γ signaling on viral RNA clearance in vivo. Viral RNA levels were examined by qRT-PCR in brains (**A**) and spinal cords (**B**) of WT (black circle and solid line), *Ifngr1^−/−^* (gray square and solid line), and *Ifng^−/−^* (white circle and black dashed line) mice (*n* = 3–8 mice per strain per time point; data are presented as the mean ± SEM; * *p <* 0.05, *** *p <* 0.001, WT vs. *Ifngr1^−/−^*; ^#^ *p <* 0.05, ^##^ *p <* 0.01, ^###^ *p <* 0.001, and WT vs. *Ifng^−/−^*, all by Tukey’s multiple comparisons test).

Clearance of viral RNA from the CNS was examined by quantifying SINV RNA in brains and spinal cords of SINV-infected WT, *Ifng^−/−^* and *Ifnrg^−/−^* mice by qRT-PCR using primers specific for the E2 gene (Figure 5). Viral RNA peaked at 3 to 5 DPI in the brains (Figure 5A) and spinal cords (Figure 5B), with comparable amounts for all mice. However, at 7 DPI, viral RNA levels were higher in the brains and at 5 and 7 DPI in the spinal cords of WT mice compared to *Ifngr1^−/−^* and *Ifng^−/−^* mice. Viral RNA levels were comparable throughout Phase 2 of infection; however, at occasional times during Phase 3, when viral RNA had reached a low-level steady state, viral RNA increased, especially in the spinal cords of *Ifng^−/−^* mice. The results indicate that while impaired IFN-γ signaling results in delayed infectious virus clearance [7,9,30], initiation of viral RNA clearance was accelerated, but the likelihood of reactivation of viral RNA synthesis late after infection was increased.

### 3.6. Effect of IFN-γ Signaling on CNS Pathology in Response to SINV Infection

**Figure 6:**
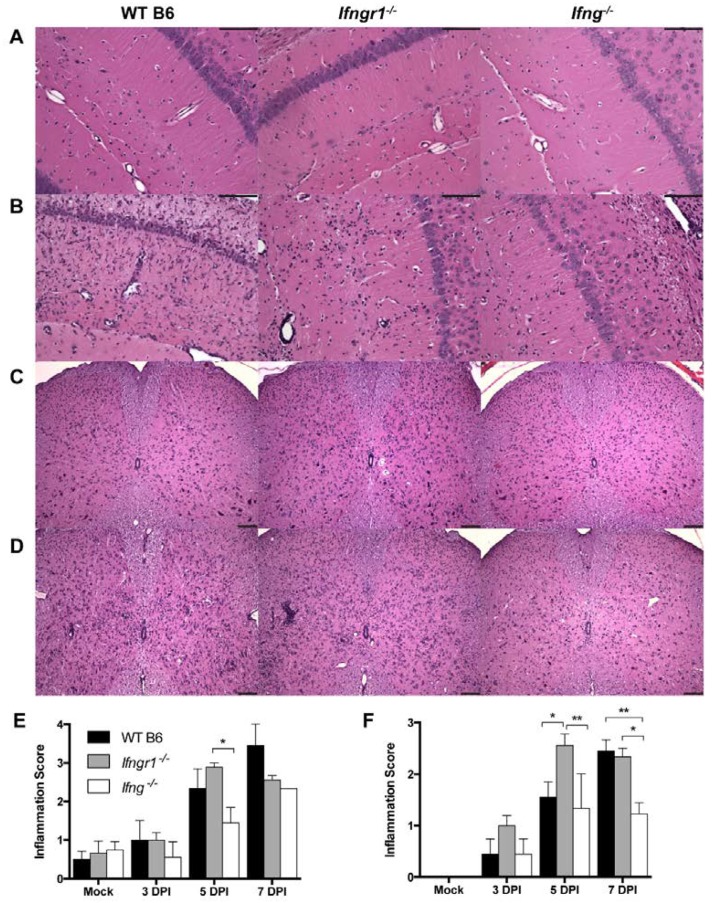
Inflammation in the brain and spinal cord of SINV-infected WT, *Ifngr1^−/−^*, and *Ifng^−/−^* mice. (**A**–**D**) Representative photomicrographs were taken of H&E-stained brain (**A**,**B**) and spinal cord (**C**,**D**) sections from mock-infected mice (**A**,**C**) and SINV-infected mice at 7 DPI (**B**,**D**) (brain = 200× magnification and spinal cords = 100× magnification, scale bar = 100 μm). (**E**,**F**) H&E sections of brains (**E**) and spinal cords (**F**) of WT (black bars), *Ifngr1^−/−^* (gray bars), and *Ifng^−/−^* (white bars) mice either mock-infected or SINV-infected at 3, 5 or 7 DPI were scored for inflammation using a four-point (brain) or three-point (spinal cord) system (data are presented as the mean score ± SEM for 3–4 mice per strain per group; * *p* < 0.05 and ** *p* < 0.01, Tukey’s multiple comparisons test).

To examine the effect of IFN-γ signaling on the inflammatory response to SINV infection, we first examined brain and spinal cord pathology of SINV-infected WT, *Ifng^−/−^* and *Ifngr1^−/−^* mice. Most of the pathological changes seen with alphavirus encephalomyelitis are associated with infiltration of immune cells [14,23,24,31,32,33]. Brains and spinal cords were examined for histopathological changes and inflammation at 0, 3, 5, and 7 DPI (Figure 6). Sporadically at 5 DPI and consistently at 7 DPI, brains from WT mice had diffuse bilateral dilation of the lateral ventricles, extending along the dorsal aspects of the hippocampi. This change was rarely seen in brains from *Ifngr1^−/−^* or *Ifng^−/−^* mice, and when present, was less severe. Compared to mock-infected control mice (Figure 6A), both perivascular cuffing and infiltration of mononuclear cells into the parenchyma in the brain were present at 7 DPI in all SINV-infected mice (Figure 6B). In the spinal cord, compared to mock-infected controls (Figure 6C), SINV-infected WT and *Ifngr1^−/−^* mice had more inflammation than *Ifng^−/−^* mice (Figure 6D). For quantitative comparison of the inflammation in brain (Figure 6E) and spinal cord (Figure 6F) at 3, 5, and 7 DPI, a scoring system was used to evaluate coded H&E-stained sections [23,24]. Inflammation steadily increased during the first week of infection, peaking at 7 DPI in WT and *Ifng^−/−^* mice and at 5 DPI in *Ifngr1^−/−^* mice. In both tissues, inflammation scores were lower in *Ifng^−/−^* mice than in WT or *Ifngr1^−/−^* mice. Minimal inflammation present in the brain but not spinal cord of mock-infected mice was likely due to trauma from the IC inoculation. 

### 3.7. The Effect of IFN-γ Signaling on Proliferation and Infiltration of Immune Cells into the CNS

**Figure 7:**
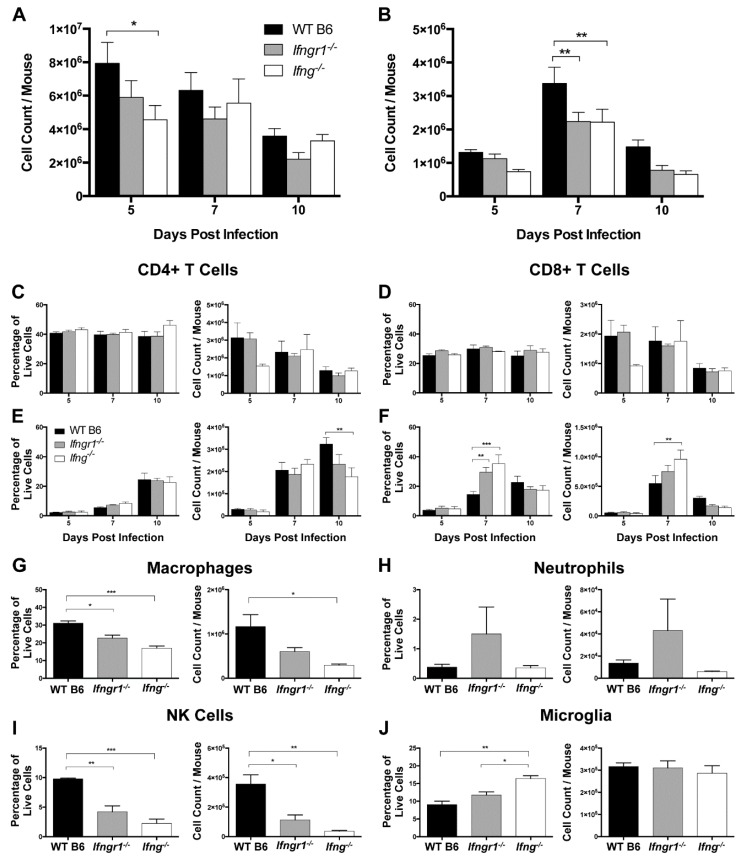
Role of IFN-γ signaling in immune cell proliferation and infiltration into the brain during SINV infection. (**A**,**B**) Total live mononuclear cells were evaluated in the CLNs (**A**) and brains (**B**) of WT (black bars), *Ifngr1^−/−^* (gray bars), and *Ifng^−/−^* (white bars) mice by trypan blue exclusion at 5, 7, and 10 DPI (*n* = 3–7 pooled mice per strain per time point from five independent experiments). (**C**–**F**) Flow cytometry was used to evaluate changes in the CLNs (**C**,**D**) and infiltration into the brain (**E**,**F**) of CD4^+^ T cells (**C**,**E**) and CD8^+^ T cells (**D**,**F**) at 5, 7, and 10 DPI. Cell data are presented as both percentage of live cells (**left graphs**) and absolute cell counts (**right graphs**) (*n* = 3–7 pooled mice per strain per time point from three independent experiments; data are presented as the mean SEM; * *p <* 0.05, ** *p <* 0.01, *** *p <* 0.001, by Tukey’s multiple comparisons test). (**G**–**J**) Flow cytometry was used to evaluate infiltration of macrophages (**G**), and neutrophils (**H**), and NK cells (**I**), and proliferation of microglia (**J**) at 7 DPI. Cell data are presented as both percentage of live cells (**left graphs**) and absolute cell counts (**right graphs**) (*n* = 4–8 pooled mice per strain per time point from three independent experiments; data are presented as the mean SEM; * *p <* 0.05, ** *p <* 0.01, and *** *p <* 0.001, by Tukey’s multiple comparisons test).

To determine the effects of IFN-γ signaling on immune cell subsets after SINV infection, cells isolated from the CLNs and brains of WT, *Ifng^−/−^* and *Ifngr1^−/−^* mice were examined by flow cytometry. In the CLNs at 5 DPI, there were more total mononuclear cells in WT mice than *Ifng^−/−^* mice (Figure 7A), but similar numbers at 7 and 10 DPI. Neither the percentage nor absolute number of CD4^+^ T cells (Figure 7C) or CD8^+^ T cells (Figure 7D) in CLNs were affected by impaired IFN-γ signaling, with a general overall decrease from 5 DPI to 10 DPI.

In contrast, WT mice had more total mononuclear cells in the brain at 7 DPI than *Ifngr1^−/−^* and *Ifng^−/−^* mice (Figure 7B). Numbers of infiltrating CD8^+^ T cells peaked at 7 DPI, while infiltration of CD4^+^ T cells occurred later with peaks at 10 DPI. WT mice had more CD4^+^ T cells than *Ifng^−/−^* mice at 10 DPI (Figure 7E), while both the percentage of cells and absolute numbers of CD8^+^ T cells were lower in brains of WT mice than *Ifngr1^−/−^* and *Ifng^−/−^* mice at 7 DPI (Figure 7F), despite the fact that WT mice had more overall brain mononuclear cells at this time (Figure 7B). Therefore, while the absence of IFN-γ signaling did not affect the proliferation of T cells in CLNs in response to SINV infection, it did affect recruitment of these cells to the site of infection in the brain.

Because WT mice have more inflammation (Figure 6) and more mononuclear cells (Figure 7B), but fewer CD8^+^ T cells (Figure 7F) in brain compared to *Ifngr1^−/−^* and *Ifng^−/−^* mice at 7 DPI, non-T cell immune cell populations were assessed. Macrophages (Figure 7G) and NK cells (Figure 7I), both as a percentage of the total live mononuclear cell population and absolute numbers, were higher in WT mouse brains compared to mice with impaired IFN-γ signaling, while neutrophils tended to be higher in *Ifngr1^−/−^* mouse brains (Figure 7H). Microglial cells as a percentage of the total brain mononuclear cell population were higher in *Ifng^−/−^* compared to WT and *Ifngr1^−/−^* mice (Figure 7J), but absolute numbers of microglia were comparable. Therefore, the larger number of mononuclear cells in the brains of WT mice at 7 DPI compared to mice with impaired IFN-γ signaling was due to infiltration of more macrophages and NK cells.

### 3.8. The Effect of IFN-γ Signaling on the Function of Brain CD4^+^ T Cells during SINV Infection

**Figure 8:**
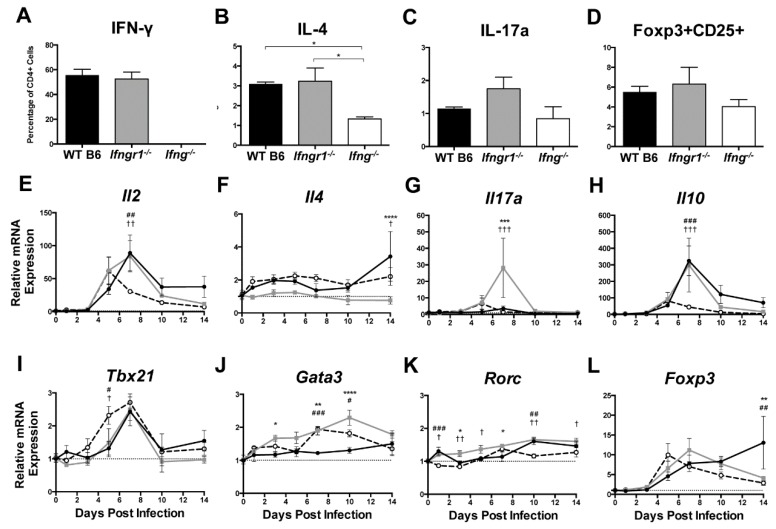
T helper cell profiles in brains of SINV-infected mice with impaired IFN-γ signaling. Flow cytometry was used to examine the percentage of CD4^+^ T cells producing IFN-γ (**A**), IL-4 (**B**), IL-17a (**C**), or expressing both Foxp3 and CD25 (**D**) in WT (black bars), *Ifngr1^−/−^* (gray bars), and *Ifng^−/−^* (white bars) mice at 7 DPI. These markers denoted Th1, Th2, Th17, and Treg cell populations, respectively (*n* = 3–7 pooled mice per strain per time point from three independent experiments; data are presented as the mean SEM; * *p <* 0.05 by Dunn’s multiple comparisons test). (**E**–**L**) Relative mRNA expression of *Il2* (**E**), *Il4* (**F**), *Il17a* (**G**), *Il10* (**H**), *Tbx21* (**I**), *Gata3* (**J**), *Rorc* (**K**), and *Foxp3* (**L**) were examined by qRT-PCR in WT (black circle and solid line), *Ifngr1^−/−^* (gray square and solid line), and *Ifng^−/−^* (white circle and black dashed line) mouse brains (*n* = 3–4 mice per strain per time point; data are presented as the mean ± SEM; dashed line indicates gene expression of 0 DPI tissue for each strain to which other time points were normalized; * *p <* 0.05, ** *p <* 0.01, *** *p <* 0.001, **** *p <* 0.0001, WT vs. *Ifngr1^−/−^*; ^#^ *p <* 0.05, ^##^ *p <* 0.01, ^###^ *p <* 0.001, WT vs. *Ifng^−/−^*, ^†^ *p <* 0.05, ^††^ *p <* 0.01, ^†††^ *p <* 0.001, and *Ifngr1^−/−^* vs. *Ifng^−/−^*, all by Tukey’s multiple comparisons test).

Because impaired IFN-γ signaling altered the numbers of CD4^+^ and CD8^+^ T cells infiltrating the brain during SINV infection, we sought to determine the effector function of these cells. Brain CD4^+^ T cells at 7 DPI were characterized further by measuring production of signature cytokines and transcription factors (Figure 8A–D) and expression of cytokine (Figure 8E–H) and transcription factor (Figure 8I–L) mRNAs associated with different T helper (Th) subsets. As expected, IFN-γ (Th1 cells) was not produced by CD4^+^ T cells in *Ifng^−/−^* mice, and the percentage of CD4^+^ T cells producing IFN-γ did not differ between WT and *Ifngr1^−/−^* mice (Figure 8A). The percentage of CD4^+^ T cells producing IL-4 (Th2 cells) was lower in *Ifng^−/−^* mice than WT or *Ifngr1^−/−^* mice (Figure 8B). Although not significant, more CD4^+^ T cells in *Ifngr1^−/−^* mice produced IL-17a (Th17 cells) compared to WT and *Ifng^−/−^* mice (Figure 8C), and the percentage of CD4^+^ T cells expressing both CD25 and Foxp3 (Tregs) trended lower in *Ifng^−/−^* mice (Figure 8D). These results show that IFN-γ signaling affects CD4^+^ T cell function during SINV infection, but impaired signaling has only a modest effect on Th subset profile.

To further evaluate Th subsets, mRNAs for four cytokines associated with specific Th profiles were examined: *Il2* for Th1 cells (Figure 8E), *Il4* for Th2 cells (Figure 8F), *Il17a* for Th17 cells (Figure 8G), and *Il10* for Tregs (Figure 8H). At 7 DPI, mRNA expression of *Il2* and *Il10* was lower in *Ifng^−/−^* mice, and expression of *Il17a* was higher in *Ifngr1^−/−^* mice. *Il4* expression did not significantly differ among strains. Expression of mRNAs for transcription factors *Tbx21* for Th1 cells (Figure 8I), *Gata3* for Th2 cells (Figure 8J), *Rorc* for Th17 cells (Figure 8K), and *Foxp3* for Tregs (Figure 8L) was also measured. While significant differences were found between strains at various time points, no major trends were identified. Therefore, CD4^+^ T cell differentiation mostly affected *Ifng^−/−^* mice with fewer Th2 and Treg cells and *Ifngr1^−/−^* mice with more Th17 cells. Modulation of Th profiles during SINV infection by IFN-γ signaling warrants further examination. 

### 3.9. The Effect of IFN-γ Signaling on the Function of CD8+ T Cells in the CNS during SINV Infection

**Figure 9:**
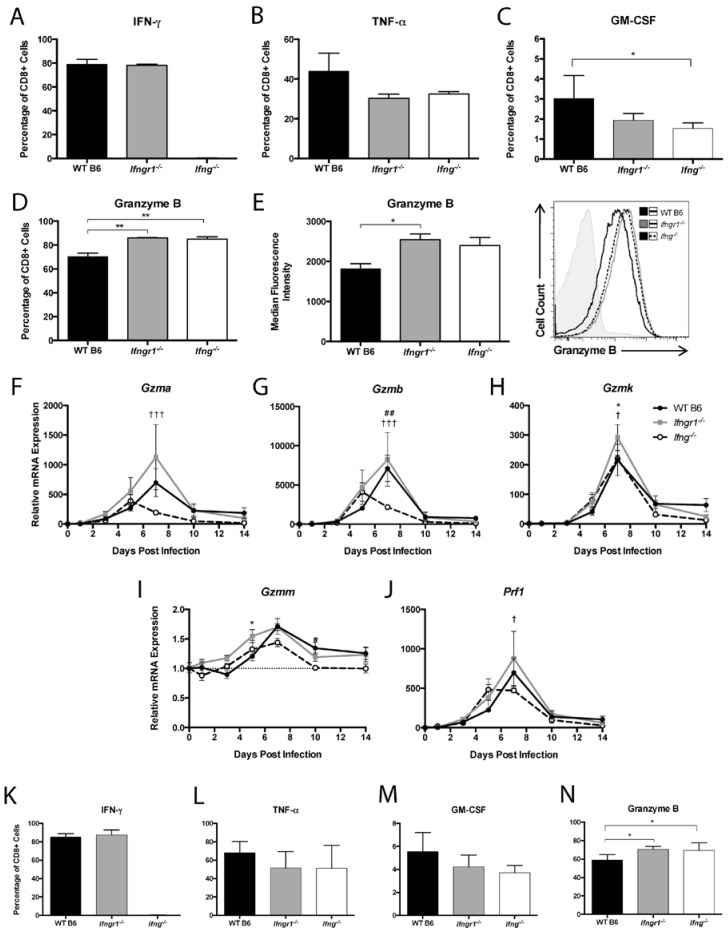
CD8^+^ T cell function during SINV infection in WT, *Ifngr1^−/−^*, and *Ifng^−/−^* mouse brains and spinal cords. (**A**–**D**) Flow cytometry was used to evaluate the percentage of CD8^+^ T cells producing IFN-γ (**A**), TNF-α (**B**), GM-CSF (**C**), and granzyme B (**D**) in the brains of WT (black bars), *Ifngr1^−/−^* (gray bars), and *Ifng^−/−^* (white bars) mice at 7 DPI. (**E**) MFI presented in graph form (**left**) and as a histogram (**right**) was used to evaluate the amount of granzyme B produced by individual CD8^+^ T cells among strains (*n* = 3–9 pooled mice per strain per time point from 3–4 independent experiments; * *p <* 0.05, ** *p* < 0.01 by Dunn’s multiple comparisons test). (**F**–**J**) Relative mRNA expression of granzyme A (**F**), granzyme B (**G**), granzyme K (**H**), granzyme M (**I**), and perforin (**J**) were examined by qRT-PCR in WT (black circle and solid line), *Ifngr1^−/−^* (gray square and solid line), and *Ifng^−/−^* (white circle and black dashed line) mouse brains (*n* = 3–4 mice per strain per time point; data are presented as the mean ± SEM; dashed line indicates gene expression of 0 DPI tissue for each strain to which other time points were normalized; * *p <* 0.05, WT vs. *Ifngr1^−/−^*; ^##^ *p <* 0.01, WT vs. *Ifng^−/−^*, ^†^ *p <* 0.05, ^†††^ *p <* 0.001, *Ifngr1^−/−^* vs. *Ifng^−/−^*, all by Tukey’s multiple comparisons test). (**K**–**N**) Flow cytometry was used to evaluate the percentage of CD8^+^ T cells producing IFN-γ (**K**), TNF-α (**L**), GM-CSF (**M**), and granzyme B (**N**) in the spinal cords of WT, *Ifngr1^−/−^*, and *Ifng^−/−^* mice at 7 DPI. (*n* = 6–9 pooled mice per strain per time point from five independent experiments; * *p <* 0.05, by Dunn’s multiple comparisons test).

To examine how IFN-γ signaling affects CD8^+^ T cell production of effector proteins at 7 DPI, IFN-γ, TNF-α, GM-CSF, and granzyme B in cells from the brains of WT, *Ifngr1^−/−^*, and *Ifng^−/−^* mice were examined by flow cytometry. CD8^+^ T cells from *Ifng^−/−^* mice did not produce IFN-γ, and the percentage of CD8^+^ T cells producing IFN-γ did not differ between WT and *Ifngr1^−/−^* mice (Figure 9A). The percentage of CD8^+^ T cells producing TNF-α was not significantly different between strains (Figure 9B), but more CD8^+^ T cells from WT mice than *Ifng^−/−^* mice produced GM-CSF (Figure 9C), while more CD8^+^ T cells from both *Ifngr1^−/−^* and *Ifng^−/−^* mice than WT mice produced granzyme B (Figure 9D). Furthermore, the granzyme B MFI of CD8^+^ T cells from *Ifngr1^−/−^* mice was also higher than WT mice (Figure 9E), indicating that individual CD8^+^ T cells in the brains of mice with impaired IFN-γ signaling produced more granzyme B than cells with intact IFN-γ signaling. Expression of granzyme A (Figure 9F) and granzyme B (Figure 9G) mRNAs in brain was lower in *Ifng^−/−^* mice compared to WT and *Ifngr1^−/−^* mice, but expression differences among strains for granzyme K (Figure 9H), granzyme M (Figure 9I), and perforin (Figure 9J) were less pronounced. 

Effector function of CD8^+^ T cells in the spinal cords of infected mice were similar to the brain in that IFN-γ-producing CD8^+^ T cells were not detected in *Ifng^−/−^* mice and were comparable between WT and *Ifngr1^−/−^* mice (Figure 9K). TNF-α (Figure 9L) and GM-CSF (Figure 9M) production by CD8^+^ T cells were not different, but the percentage of CD8^+^ T cells producing granzyme B (Figure 9N) was lower in WT mice than mice with impaired IFN-γ signaling. These findings indicate that IFN-γ signaling not only inhibits infiltration of CD8^+^ T cells into the CNS, but also inhibits CD8^+^ T cell synthesis of granzyme B.

### 3.10. Effect of IFN-γ Signaling on CD8^+^ T Cell and NK Cell Degranulation and Cytotoxic Function during SINV Infection

**Figure 10:**
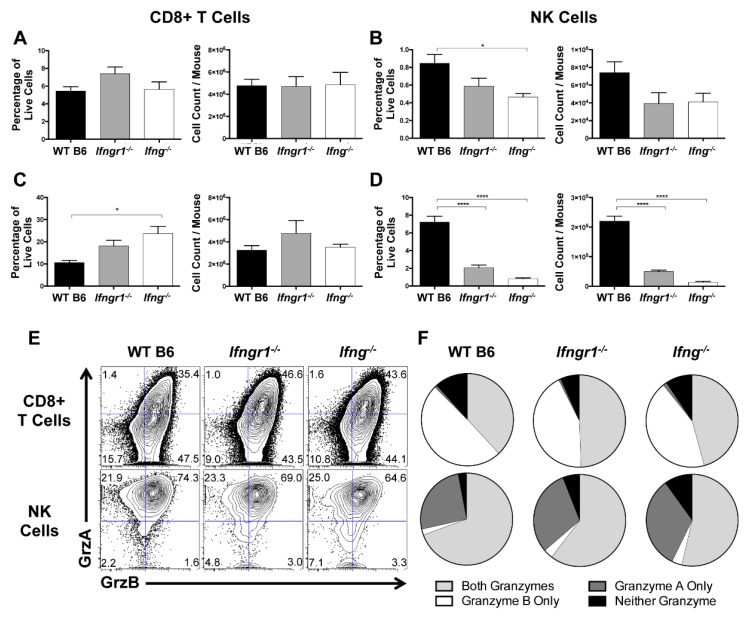
Effect of IFN-γ on CD8^+^ T cell and NK cell degranulation and cytotoxicity. (**A**–**D**) Flow cytometry was used to examine the percentage of CD8^+^ T cells (**A**,**C**) or NK cells (**B**,**D**) expressing CD107a, indicative of degranulation, in CLNs (**A**,**B**) and brains (**C**,**D**) of WT (black bars), *Ifngr1^−/−^* (gray bars), and *Ifng^−/−^* (white bars) mice at 7 DPI (*n* = 6–9 pooled mice per time point from four independent experiments; data are presented as the mean ± SEM; * *p <* 0.05, **** *p <* 0.0001 by Dunn’s multiple comparisons test). (**E**,**F**) Contour plots (**E**) show the gating, denoted by blue lines, of CD8^+^ T cells (**top row of plots**) and NK cells (**bottom row of plots**) for granzyme A and granzyme B production in the brain at 7 DPI. Boolean gating (**F**) was used to determine whether stimulated CD8^+^ T cells (**top row**) and NK cells (**bottom row**) were producing both granzymes A and B (light gray), granzyme A only (dark gray), granzyme B only (white), or neither of the granzymes (black) at 7 DPI (*n* = 6–9 pooled mice per time point from three independent experiments; data are presented as the mean).

CD8^+^ T cells and NK cells primarily exert their cytolytic effects through secretion of cytotoxic granzymes that kill target cells [34]. Because infiltration of CD8^+^ T cells and NK cells into the brain were differentially regulated by IFN-γ signaling, the extent of degranulation, as identified by CD107a expression [35], and granzyme production were examined in cells from the CLNs and brains of WT, *Ifngr1^−/−^*, and *Ifng^−/−^* mice at 7 DPI. In CLNs, CD107a expression by CD8^+^ T cells was minimally affected by IFN-γ signaling (Figure 10A). In brain, the percentage of CD8^+^ T cells expressing CD107a was higher in *Ifng^−/−^* mice than WT mice, but absolute numbers were comparable (Figure 10C). In contrast, WT mice had more degranulated NK cells in the CLNs (Figure 10B) and the brain (Figure 10D) than either *Ifngr1^−/−^* or *Ifng^−/−^* mice. Therefore, IFN-γ signaling affected the cytotoxic function of local CNS NK cells and CD8^+^ T cells during SINV infection, but in opposite directions.

Granzymes A and B are the primary granzymes involved in cytotoxicity of CD8+ T cells and NK cells [36], so we next identified the granzymes produced in the brain at 7 DPI during SINV infection and the effect of IFN-γ signaling using Boolean gating (Figure 10E,F). Approximately 35 to 40% of CD8^+^ T cells and 65 to 70% of NK cells in the brain produced both granzyme A and B. However, if producing only one granzyme, CD8^+^ T cells preferentially produced granzyme B, while NK cells preferentially produced granzyme A (Figure 10F). Additionally, granzyme production was lower in CD8^+^ T cells but higher in NK cells of WT mice compared to mice with impaired IFN-γ signaling. Taken together, these data suggest that IFN-γ promotes NK cell cytotoxicity but suppresses the cytotoxic potential of CD8^+^ T cells in the brain during SINV infection.

### 3.11. The Effect of IFN-γ Signaling on Establishment of CD8^+^ Tissue-Resident Memory (T_RM_) Cells in the CNS after SINV Infection

**Figure 11:**
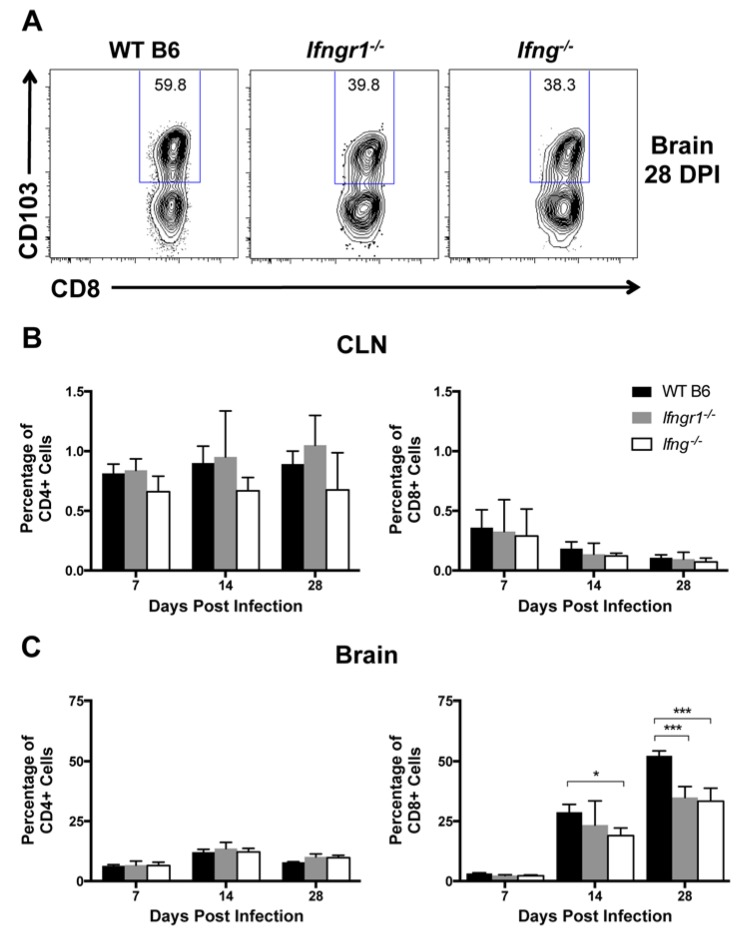
Effect of IFN-γ signaling on T_RM_ cell populations. Flow cytometry was used to examine T_RM_ cell populations by gating, denoted by blue frames, around CD103^+^ cells (**A**) at 7, 14, and 28 DPI in the CLNs (**B**) and brains (**C**) of WT (black bars), *Ifngr1^−/−^* (gray bars), and *Ifng^−/−^* (white bars) mice, and results are presented as a percentage of CD4^+^ (**left graphs**) and CD8^+^ T cells (**right graphs**) (*n* = 2–7 pooled mice per strain per time point from three to four independent experiments; data are presented as the mean ± SEM; * *p <* 0.05, *** *p <* 0.001 by Tukey’s multiple comparisons test).

Because long-term control of viral RNA is likely required to prevent reactivation of virus production and relapse of clinical disease, immune cells remain in the CNS long term after SINV infection [5]. Tissue-resident memory (T_RM_) cells are a subset of memory T cells that do not circulate, but instead permanently remain at sites of infection [37]. CD103^+^ T_RM_ cells were assessed in CLNs and brains in WT, *Ifngr1*^−/−^, and *Ifng*^−/−^ mice at 7, 14, and 28 DPI to determine a role for IFN-γ in their development, maintenance, and survival (Figure 11A). Very few CD4^+^ or CD8^+^ T_RM_ cells were present in CLNs of SINV-infected mice at any time after infection (Figure 11B). Higher percentages of CD4^+^ T cells in the brain were T_RM_ cells, but they did not change over time or differ among mouse strains (Figure 11C, left panel). However, percentages of CD8^+^ T_RM_ cells in the brain increased over time (Figure 11C, right panel) and were more abundant in the brains of WT mice than *Ifngr1*^−/−^ mice at 28 DPI and *Ifng*^−/−^ mice at 14 and 28 DPI. The results suggest that IFN-γ signaling promotes the development of CD8^+^ T_RM_ cells in the brain during SINV infection and affects their presence following infectious virus clearance.

## 4. Discussion

IFN-γ is an important determinant of the outcome of virus infections of the CNS, with both induction of antiviral genes and regulation of the immune response to infection. Previous studies have shown that IFN-γ facilitates clearance of infectious virus from spinal cord neurons during SINV infection [7], and in vitro*,* IFN-γ treatment inhibits replication of SINV in neurons through the Jak/Stat signaling pathway [16,38]; however, the antiviral genes induced were not identified. The current study showed that mRNAs for Gbp2 and Irgm1 GTPase proteins associated with autophagy were highly induced by IFN-γ signaling in SINV-infected neurons as well as in the CNS of SINV-infected mice where multiple cells may be responding to secreted IFN-γ. Autophagy can decrease virus replication by destroying virus or viral replication components, including alphaviruses, or by delivering them to endosomes for Toll-like receptor (TLR) induction of the innate immune response [39,40,41]. RNA viruses, including the alphavirus chikungunya virus (CHIKV), target IRGM to promote virus replication [42,43]. Another antiviral protein system highly induced by IFN-γ signaling during SINV infection of neurons was the 2′-5′-oligoadenylate synthetase (OAS) family, a pathway that affects replication of several neurotropic viruses, including rabies virus, canine distemper virus, West Nile virus (WNV), and JEV [29,44,45,46]. Two other antiviral genes usually more robustly induced by type I than type II IFNs, *Rsad2* and *Zc3hav1*, were induced later and to lesser extents. *Rsad2*-encoded viperin can impair budding of enveloped viruses [47,48,49] and alphavirus and flavivirus replication and assembly [50,51,52,53]. *Zc3hav1* encodes zinc-finger antiviral protein (ZAP) or poly(ADP-ribose) polymerase-13 (PARP13), an RNA-binding protein that recruits RNA decay factors to degrade viral RNA and inhibit viral RNA translation [54,55,56,57,58]. However, the specific role for these ISGs in IFN-γ-mediated virus clearance from neurons will require further study.

The current studies show that the in vivo role of IFN-γ in pathogenesis of SINV-induced encephalomyelitis is complex. Although IFN-γ is important for clearance of infectious virus, particularly from spinal cord neurons [7,30], and was sufficient for viral RNA clearance in neurons in vitro, IFN-γ delayed clearance initiation of viral RNA from both the brain and spinal cord in mice (Figure 5), suggesting an extra neuronal effect that secondarily affects viral RNA clearance. IFN-γ signaling improved recruitment of NK cells but differentially affected the recruitment of T cells, with more CD4^+^ T cells and fewer CD8^+^ T cells infiltrating the brains of SINV-infected WT mice than *Ifngr1*^−/−^ or *Ifng*^−/−^ mice. This modification of the local T cell response potentially explains the differential initiation of RNA clearance among mice with intact and impaired IFN-γ signaling, leading us to further evaluate the composition and functionality of these T cells. 

CD4^+^ T cells primarily exert their effects via cytokine secretion, which identify Th subsets that influence the differentiation and activation of other immune cells [59]. Treg cells, defined by expression of both Foxp3 and CD25, tended to be lower in *Ifng*^−/−^ mouse brains, and mRNA expression of the regulatory cytokine IL-10 was significantly lower compared to WT and *Ifngr1*^−/−^ mice at 7 DPI. Th17 cells have been associated with fatal encephalomyelitis and virus persistence during infection with several viruses, such as SINV NSV, TMEV, and JHMV, especially in the absence of IFN-γ [12,60,61,62]. *Ifngr1*^−/−^ mice expressed more *Il17a* mRNA and trended toward increased IL-17a production by CD4^+^ T cells compared to WT and *Ifng*^−/−^ mice. Similar results were seen in *Ifngr1*^−/−^ mice infected with SINV NSV and suggest preferential expansion of Th17 cells [30]. Neutrophils, also associated with a Th17 profile [63], were also increased in brains of *Ifngr1*^−/−^ mice. These results show that IFN-γ signaling affects CD4^+^ T cell function, although major differences in Th profile were not seen, and reiterates the differences between mice lacking IFN-γ production and mice with impaired receptor function in the immune response to SINV infection.

In our mouse model of alphavirus encephalomyelitis, fewer CD8^+^ T cells infiltrated the CNS in WT mice with intact IFN-γ signaling, an effect similar to the suppression of the CD8^+^ T cell response to Friend virus infection observed in association with lactate dehydrogenase virus-induced IFN-γ [64]. As with NK cells, CD8^+^ T cells primarily exert their effector function against virus infections through cytotoxic activity and produce granzymes and perforin for delivery through granule exocytosis to activate caspases and induce target cell apoptosis [34]. A clear role for NK cells in pathogenesis of alphavirus encephalomyelitis has not been identified [65,66] and, in the current studies, greater numbers of NK cells and granzyme A expression in WT mice did not offset the diminished CD8^+^ T cell response. Specific targeting of infected cells by CD8^+^ T cells is achieved through direct contact with an infected cell expressing MHC Class I molecules. Neurons have a limited capacity for MHC class I expression [2,67,68,69,70], but CD8^+^ T cells can directly engage virus-infected neurons [71], and clearance of WNV, JEV, and LCMV from the CNS is dependent on the granzyme/perforin pathway [72,73,74]. Perforin production is decreased in the brains of WT mice compared to *Ifngr1*^−/−^ and *Ifng*^−/−^ mice during SINV NSV infection [30], and perforin-mediated effector function is impaired during JHMV infection [75] However, IFN-γ promotes granzyme B production during experimental coronavirus retinopathy [76]. Therefore, the effect of IFN-γ on the granule exocytosis pathway during CNS infection appears to be virus-specific, and the mechanisms by which IFN-γ influences granzyme production during SINV infection remain to be understood.

It is becoming increasingly understood that granzymes have non-cytotoxic as well as cytotoxic roles [36,77,78,79]. For instance, CD8^+^ T cells can inhibit reactivation of HSV-1 in neurons via a non-cytolytic mechanism [80,81,82]. Mouse granzyme K induces macrophages to release IL-1β during LCMV infection [83], and granzyme substrates with direct antiviral activity, either viral proteins or host cell proteins essential for virus replication, have also been identified. Granzymes A, H, and M cleave viral proteins important for replication of Moloney mouse leukemia virus, adenovirus, and human cytomegalovirus [84,85,86]. Granzymes B and H both cleave the RNA-binding protein La, which is important in viral RNA metabolism for several viruses [87,88,89,90]. hnRNP K, which modulates viral RNA replication of CHIKV, enterovirus 71, dengue virus, and HIV-1 [91,92,93,94] and interacts with SINV nsp2 and subgenomic mRNA [95,96], is a substrate for granzyme B. Silencing of hnRNP K or disruption of hnRNP-vRNA binding decreases SINV RNA replication in vitro [96,97]. Therefore, we postulate that the increased granzyme B produced by CD8^+^ T cells of *Ifngr1*^−/−^ and *Ifng*^−/−^ cleaves one or more cellular proteins important for SINV RNA replication or stability to accelerate noncytotoxic viral RNA clearance from the brain and spinal cord. Further studies regarding viral RNA clearance, the noncytotoxic roles of granzymes during SINV infection, and regulation by IFN-γ, are warranted.

Persistence of viral RNA after CNS infection is common [5,10,98,99,100,101,102] and presents the potential for virus reactivation and relapse of disease. Indeed, during Phase 3 of infection, transient increases in viral RNA were seen in SINV-infected mice, particularly in *Ifng*^−/−^ *mice*. Therefore, prevention of virus reactivation is likely achieved through continued presence of immune cells at the previous site of infection [103,104]. Over the course of infection, the percentage of CD8^+^, but not CD4^+^, T cells expressing CD103, a marker for T_RM_ cells in the brain, increased. As permanent residents at sites of previous infection, T_RM_ cells provide a rapid response to pathogen reactivation [105,106,107]. IFN-γ produced by CD4^+^ T cells is required for generating CD8^+^ T_RM_ cells in the lung after influenza virus infection [108], and rapid clearance of LCMV by brain T_RM_ cells depends on IFN-γ signaling and cytotoxic granule release [107]. Brains of SINV-infected mice defective in IFN-γ signaling had both fewer CD4^+^ T cells and CD8^+^ T_RM_ cells than WT mice, indicating a need for IFN-γ to promote the residence of T_RM_ cells in the brain after infection. 

In conclusion, IFN-γ signaling has both positive and negative effects on SINV clearance and control. IFN-γ facilitates infectious virus clearance through direct antiviral effects on infected neurons and inducing production of B cell-attracting chemokines that fosters local production of anti-SINV antibody [9]. However, IFN-γ impairs viral RNA clearance, possibly by suppressing the CD8^+^ T cell response in the CNS and granzyme B production. Finally, IFN-γ promotes the development of CD8^+^ T_RM_ cells in the brain, likely helping prevent reactivation of persistent virus. Better understanding of the complicated interplay between the virus and host immune system in inducing pathology and promoting virus clearance is critical to developing effective therapies.
